# Ethical oversight of AI-driven paediatric trials: a proactive, risk-sensitive interim review model

**DOI:** 10.3389/fdgth.2026.1782692

**Published:** 2026-03-27

**Authors:** Chih-Shung Wong, Tsui-Wen Hsu

**Affiliations:** 1 Department of Anesthesiology, Cathay General Hospital, Taipei, Taiwan; 2 Department of CGHIRB, Cathay General Hospital, Taipei, Taiwan; 3 Institute of Medicine, Superintendent Office and CGHIRB, Cathay General Hospital, Taipei, Taiwan; 4 School of Nursing, College of Medicine, Yuanpei University of Medical Technology, Hsinchu, Taiwan; 5 School of Nursing, College of Medicine, Chang Jung Christian University, Tainan, Taiwan

**Keywords:** artificial intelligence, digital health, ethical oversight, institutional review boards, interim review, pediatric clinical trials, research ethics, risk-sensitive governance

## Abstract

**Background:**

Artificial intelligence (AI)-driven paediatric trials pose novel challenges for institutional review boards (IRBs), as traditional annual continuing review frameworks are often inadequate for evolving algorithmic and data-related risks. International and national regulations provide only limited guidance on how to design proactive, risk-sensitive interim oversight mechanisms for such research.

**Objective:**

To develop and illustrate a risk-sensitive interim review model that strengthens participant protection and procedural fairness in AI-enabled paediatric research.

**Methods:**

A conceptual normative analysis was conducted, integrating four ethical principles—protection, proportionality, respect for autonomy and assent, and procedural justice—with international guidelines [International Conference on Harmonisation–Good Clinical Practice ICH-GCP, Council for International Organizations of Medical Sciences (CIOMS), and the Declaration of Helsinki] and Taiwanese regulations. From this synthesis, a five-component proactive interim review model was developed. To illustrate the model's practical application and feasibility, a Taiwanese IRB-mandated interim review of an AI-assisted pediatric speech-therapy trial (*n* = 100, aged 3-7 years) is presented as a worked example rather than empirical data collection.

**Results:**

The model comprises five interlocking components: (1) scheduled, risk-based interim reviews and audits; (2) structured deviation-triggered response procedures; (3) mechanisms for re-consent and ongoing communication; (4) continuous ethics and protocol training; and (5) transparent, auditable documentation and IRB-investigator communication. Application of the proposed model to the Taiwanese worked example illustrates how a structured, risk-sensitive interim review process can support the identification of informed-consent and eligibility-screening deviations, facilitate targeted corrective training, and promote routine documentation monitoring.

**Conclusions:**

A proactive, risk-sensitive interim review model can support IRBs in shifting from reactive annual oversight to continuous, adaptive governance aligned with AI-specific risk profiles. The model offers a transferable, principle-based template for strengthening ethical oversight of AI-driven pediatric trials across diverse regulatory and cultural settings.

## Introduction

Ethical oversight of pediatric clinical research is grounded in well-established principles of protection, proportionality, respect for autonomy and assent, and justice (1, 2). In AI-augmented pediatric trials, the duty of protection requires not only minimizing physical and psychological risks, but also addressing data-related and algorithmic harms that may not be immediately visible to participants or clinicians (3, 4). Proportionality demands that oversight intensity track the level and nature of risk, which in AI trials may change over time as models are retrained, updated, or deployed across new populations (4, 5). Respect for autonomy and for children's developing capacities underpins robust parental permission and, where appropriate, child assent, as well as meaningful opportunities to revisit consent when risk profiles or study procedures materially change (6, 7). Justice further requires fair selection of participants, consistent application of inclusion and exclusion criteria, and avoidance of systematic disadvantage or bias related to algorithm design or deployment (8, 9).

International guidelines, such as the ICH-GCP) (10), the CIOMS (11) and the Declaration of Helsinki (12) require ongoing review and protocol deviation management, including periodic reassessment of risk-benefit ratios, reporting of serious or systemic non-compliance, and the possibility of modifying or suspending trials to protect participants. ICH-GCP E6(R3) specifically emphasizes risk-based and proportionate approaches to clinical trial oversight (13), while ICH E11(R1) provides specific guidance for pediatric populations, recognizing their unique vulnerabilities and need for enhanced protections (14). National frameworks, such as Taiwan's Human Subject Research Act (Ministry of Health and Welfare 2011) and local IRB regulations, similarly authorize review boards to require interim reports, audits, and corrective action where needed to maintain ethical and legal standards (15). These instruments clearly assign responsibilities to sponsors, investigators, and IRBs, but they typically specify how interim oversight should be structured for low-risk technologies such as AI, leaving substantial discretion to local institutions.

AI-driven interventions present unique regulatory challenges that interact with existing norms and regulatory frameworks (16, 17). AI algorithms may exhibit performance drift over time due to changes in patient populations, clinical practice patterns, or data distributions—a phenomenon that can lead to emerging algorithmic bias even when models initially performed equitably across demographic subgroups (18, 19). Downstream uses of data, including secondary analyses or model retraining, may exceed the initial expectations set at the time of informed consent (20, 21).These characteristics exacerbate long-standing concerns about the transparency, interpretability, and accountability of clinical decision-making, particularly in pediatrics, where participants often struggle to understand or question the role of AI in their care (22, 23).

At the same time, empirical experience with digital and AI-enabled trials suggests that many protocol deviations still stem from human and organizational factors—such as incomplete or incorrectly signed informed consent forms, inconsistent application of inclusion criteria, and inconsistencies in documentation—rather than malfunctions in the AI systems themselves (24, 25). Therefore, effective interim regulation must address both the risks of the digital age and the persistent human vulnerabilities in research practice.

Recent commentary and regulatory discussions on digitized and AI-assisted research highlight emerging expectations regarding ethical oversight. These include strengthening the review of electronic and remote informed consent processes, implementing specific privacy and data security safeguards, and insisting on the auditability and interpretability of AI tools used in research (26, 27). International guidance increasingly emphasizes the importance of risk-based monitoring and adaptive review cycles that address evolving technological and clinical risks, rather than relying solely on fixed annual reviews (28, 29). There is also growing recognition of the need for ongoing, context-based training for researchers and staff covering digital informed consent, data management, and appropriate participant recruitment in technology-enabled research (26, 27, 30).

Despite these advances, existing guidelines offer limited specific guidance on designing structured interim review mechanisms for AI-driven pediatric trials. Current international and national regulations outline the general responsibilities of IRBs, including protecting vulnerable participants, ensuring oversight is risk-sensitive, ensuring effective informed consent, and addressing bias (31, 32). Principal investigators (PIs) often lack clarity about IRB expectations regarding regular interim reviews, which types of deviations should automatically trigger audits or site visits, and how to appropriately re-obtain informed consent and provide corrective training when issues are identified. IRBs, in turn, often rely solely on written investigator reports, leading to inconsistencies in how different protocols are monitored and in how deviations are handled. This variability compromises procedural fairness and leaves potential gaps in participant protection.

In this Policy and Practice Review, the paper addresses this gap by developing a proactive, risk-sensitive interim review model specifically tailored to AI-driven paediatric trials. The model translates high-level ethical principles and regulatory expectations into five operationalized components that IRBs can implement, adapt, and refine according to local legal, resource, and cultural contexts. Rather than relying on empirical data collection, this policy analysis synthesizes core ethical obligations in pediatric research protection, proportionality, respect for autonomy and assent, and procedural justice—and integrates them with relevant international regulatory frameworks (ICH-GCP, CIOMS, Declaration of Helsinki) and Taiwanese national regulations governing continuing review and protocol deviation management. Drawing on this normative synthesis, the paper develops and articulates the five-component interim review model. To illustrate how the model operates in practice and to explore its feasibility, strengths, and limitations in a concrete institutional setting, a Taiwanese IRB-mandated interim review of an AI-assisted paediatric speech-therapy trial is presented as a worked example. This case serves an illustrative purpose to demonstrate the model's application rather than as an empirical validation of the framework's effectiveness.

### Normative methodology

This paper employs a conceptual normative analysis to develop a principle-based interim review model for AI-driven pediatric trials. Rather than drawing on empirical data collection, the analysis integrates four core ethical principles—protection, proportionality, respect for autonomy and assent, and procedural justice—with international and national regulatory frameworks, including ICH-GCP, CIOMS, the Declaration of Helsinki, and relevant Taiwanese regulations. These sources are treated as the primary “data” for analysis, and the model is derived through critical interpretation, comparison, and functional mapping of their ethical and regulatory expectations. From this integration of ethical principles and regulatory frameworks, a functional five-component model of risk-sensitive interim review was constructed. Each component corresponds to a specific ethical function (e.g., proportional risk-based oversight, structured responses to serious deviations, renewed consent when risk profiles change, continuous ethics training, and transparent documentation and communication). The Taiwanese AI-assisted pediatric speech-therapy trial is then introduced as an illustrative worked example to demonstrate how the conceptually derived model can be applied in practice. The case is used to test the practical coherence and feasibility of the proposed components, but it does not serve as empirical validation of their effectiveness. Future empirical research is needed to evaluate the model's impact on protocol deviations, consent quality, and participant protection in diverse settings.

### A proactive, risk-sensitive interim review model

The proposed proactive, risk-sensitive interim review model comprises five interlocking components designed to shift IRBs from reactive annual oversight to continuous, adaptive governance. Each component addresses specific gaps identified in current AI pediatric trial oversight while remaining adaptable to diverse institutional contexts and resource levels. Together, these components operationalize the ethical principles of protection, proportionality, respect for autonomy and assent, and procedural justice in the context of evolving AI-related risks and persistent human-factor vulnerabilities.

### Component 1: scheduled, risk-based interim reviews and audits

In this model, the IRB requires scheduled interim reviews of AI-driven paediatric trials, and on-site audits where appropriate, with the timing and intensity of review calibrated to the level and type of risk, rather than relying solely on annual continuing review. This risk-based scheduling helps the IRB detect consent errors, misapplication of eligibility criteria, and documentation gaps before they compromise participant safety, fairness, or data integrity (33, 34). To enable consistent IRB application of these AI-specific risks, Table 1 operationalizes each risk type with precise definitions, quantitative. thresholds, and detection mechanisms. AI-specific risks that warrant heightened interim oversight include:

1. **Algorithmic bias and fairness drift:** AI models may produce systematically biased results due to flawed design, underrepresented training data, or evolving performance disparities across demographic subgroups over time. Unlike traditional interventions with stable risk profiles, AI systems can develop new biases post-deployment as data distributions shift or as models are retrained (35–37).
2. **Data drift and concept drift:** Model performance may degrade when patient populations, clinical practices, or data characteristics change temporally or geographically, leading to prediction errors or miscalibration that affect participant safety and trial validity (38, 39).
3. **Transparency and interpretability deficits:** Complex AI models often operate as “black boxes,” making it difficult for clinicians, participants, and IRBs to understand how decisions are made, which complicates informed consent and accountability (40, 41).
4. **Model updates and retraining:** Changes to AI algorithms, including software updates or incorporation of new training data, constitute protocol modifications that may introduce unanticipated risks and require prospective IRB review (42, 43).
5. **Privacy and data security vulnerabilities:** AI-driven trials often involve continuous data collection, remote monitoring, and secondary data uses that exceed initial consent expectations, raising heightened privacy concerns, particularly for vulnerable pediatric populations (44, 45).

**Table 1:** Risk typology: operational definitions and detection thresholds.

Risk type	Definition	Detection method	Threshold
Algorithmic bias	>5% performance differential between demographic groups	Quarterly stratified accuracy reporting	1. Expedited review if exceeded.
Data drift	>10% decrease in sensitivity/specificity from baseline	Continuous monitoring with automated alerts	Expedited review within 7 days
Transparency deficit	Lack of explainability features (SHAP, LIME, attention)	Protocol audits, comprehension surveys	Re-training if >20% don't understand
Model updates	ANY change not pre-approved in protocol	Version control logs, 24 h notification	Immediate notification, revert if unauthorized
Privacy breach	ANY unauthorized access or data breach	Security incident reporting, annual audits	24 h notification, expedited review

The operational definitions and thresholds in Table 1 are synthesized from FDA/EMA guidance on AI/ML validation and performance monitoring (16–18), algorithmic fairness and bias detection literature (35–37), data drift and model performance standards (38, 39), transparency and explainability frameworks (40, 41), and the SPIRIT-AI extension for AI clinical trial protocols (13). The 5% threshold for algorithmic bias reflects FDA recommendations for stratified performance evaluation across demographic subgroups to detect clinically meaningful disparities. The 10% threshold for data drift aligns with established criteria indicating the need for model recalibration. The 20% threshold for transparency deficits reflects comprehension assessment standards from informed consent research. Detection mechanisms are derived from emerging best practices in AI clinical trial oversight. Institutions should adapt these thresholds to local regulatory requirements, trial risk profiles, and institutional capacity while maintaining core protective functions.

Risk stratification and review frequency: IRBs should assign trials to risk categories using the AI Risk Stratification Matrix (Table 2), which determines interim review frequency based on trial characteristics. High-risk AI-enabled pediatric trials—those involving adaptive algorithms without established safety data, invasive procedures, vulnerable age groups, or novel AI applications—warrant quarterly or semi-annual interim reviews rather than an annual-only review. Moderate-risk trials may require semi-annual or annual reviews, while low-risk observational studies with validated AI tools may proceed with annual oversight supplemented by event-triggered reviews.

**Table 2:** AI risk stratification matrix for pediatric clinical trials.

Risk category	Trial characteristics	Interim review frequency	Oversight mechanisms
High Risk	1. Vulnerable pediatric age groups (<5 years) 2. Adaptive/continuously learning algorithms without established safety data, Invasive procedures combined with an AI decision support 3. Novel AI applications without regulatory precedent- 4. AI systems with documented bias in external validation	Quarterly or semi-annual	1. Mandatory on-site audits—real-time performance monitoring 2. Stratified results reporting based on demographic characteristics 3. Establishment of a Data Safety Management Team (DSMP) or Data Safety Oversight Board (DSMB) 4. Enhanced informed consent procedures and the establishment of mechanisms to trigger re-informed consent
Moderate Risk	1. School-age children (5–12 years) with parental oversight 2. Validated AI tools applied to new pediatric populations 3. Non-invasive AI-assisted interventions 4. AI systems with established safety data in related contexts- 5. Algorithms with fixed parameters (no retraining) 6. Bias monitoring reports-Targeted site visits if deviations are detected, 7. Standard consent with annual renewal	Semi-annual or annual	1. Remote documentation audits 2. Performance metric reporting 3. Targeted site visits if deviations are detected 4. Standard consent with annual renewal
Low Risk	1. Adolescents (13 + years) with capacity for assent 2. Observational studies using validated AI analytics- 3. AI tools approved for clinical use applied to research questions 4. De-identified data analysis with approved AI models and no algorithm modifications during trial	Annual	1. Document review at continuing review- 2. Event-triggered review only 3. Standard IRB oversight- Simplified reporting requirements

The risk stratification matrix Table 2 synthesizes established frameworks including ICH-GCP guidance (10, 11), risk-based monitoring literature (48, 49), and emerging FDA/EMA guidance on AI in clinical trials (16–18). The SPIRIT-AI extension (13) provides specific guidance for designing AI trial protocols. Age-based vulnerability stratification reflects well-established pediatric ethics principles (1, 2, 6, 7). AI-specific risk factors—including adaptive algorithms, novel applications without regulatory precedent, and documented algorithmic bias—reflect recent FDA/EMA frameworks emphasizing proportionate oversight.

Similar concerns about limited post-approval oversight and the need for more systematic, risk-based ethics monitoring have been documented in empirical work on ethics committee monitoring of approved studies and on-site review (46, 47). Risk-based monitoring toolboxes developed for clinical trial conduct likewise demonstrate growing international consensus that oversight intensity should be explicitly mapped to study-specific risks (48, 49), a principle this model adapts to the ethical review and interim governance of AI-enabled pediatric trials.

### Component 2: structured deviation-triggered response

Certain serious or recurrent deviations automatically trigger a structured response from the IRB. Rather than relying on *ad hoc* negotiation with investigators, predefined deviation thresholds initiate a graduated escalation pathway that may include targeted queries, mandatory corrective action plans, focused audits or on-site visits, temporary recruitment holds, or, in severe cases, suspension of the trial until risks are adequately addressed (50, 51).

AI-specific deviation triggers requiring immediate IRB notification and structured review include any algorithmic changes implemented without prospective IRB approval, such as updates to the AI model, algorithm, or software version used in the trial (52); unanticipated AI performance issues, including systematic prediction errors, calibration failures, or performance degradation across demographic subgroups that may materially alter the risk–benefit profile (38); and privacy breaches or unauthorized data use, encompassing incidents of unauthorized access to participant data, unapproved secondary analyses, or failure to implement required data security safeguards (53). In addition to these AI-specific triggers, standard deviation criteria applicable to all clinical trials remain operative, including informed consent errors (e.g., three or more improperly executed consent forms, missing parental signatures, or use of non–IRB-approved consent language) (54, 55); eligibility violations involving enrollment of participants who fail to meet inclusion criteria or meet exclusion criteria (54, 55); and recurring documentation deficiencies, such as repeated failures to maintain required study records, eligibility documentation, or adverse event logs (55).

To operationalize these deviation triggers and ensure consistent IRB responses, Table 3 specifies trigger thresholds, mandatory IRB actions, and response timelines for both AI-specific and standard protocol deviations.

**Table 3:** Protocol deviation triggers and IRB response requirements for AI-enabled pediatric trials.

Deviation type	Trigger threshold	Mandatory IRB actions	Response timeline
AI algorithm change	1. Any modification to the AI model, software version, training data, or decision logic not pre-approved by IRB	1. Immediate suspension of enrollment 2. Full board review of modification 3. Requirement for prospective approval 4. Assess need for participant re-consent	Within 7 calendar days
Algorithmic performance issues	1. Accuracy drop >10% from validation baseline- 2. Systematic prediction errors in any demographic subgroup 3. Calibration failure	1. Mandatory performance audit 2. Demographic-stratified analysis 3. Corrective action plan 4. Consider trial suspension pending resolution	Within 7 calendar days
Informed consent errors	1. ≥3 improperly signed consent forms 2. Any missing parental signature 3. Use of outdated consent version	1. Site visit or focused audit- 2. Review of all consent documentation 3. Mandatory retraining for study staff 4. Re-consent affected participants	Within 10 calendar days
Eligibility violations	1. Any participant enrolled who does not meet the inclusion criteria or meets the exclusion criteria	1. Full review of screening procedures 2. Audit of all enrolled participants 3. Corrective action plan 4. Retraining on eligibility criteria	Within 10 calendar days
Privacy/data security breach	1. Unauthorized data access- 2. Unapproved secondary analysis 3. Failure to implement required safeguards	1. Immediate investigation 2. Participant notification per regulations 3. Enhanced security measures 4. Regulatory reporting	Within 24 h
Recurring documentation gaps	1. ≥5 instances of missing required 2. Documentation Pattern of incomplete records	1. Mandatory retraining 2. Enhanced monitoring	Within 10–14 calendar days

The deviation triggers in Table 3 are derived from international regulatory frameworks, including ICH-GCP guidance (10, 11), FDA/EMA regulation (16–18), institutional IRB policies, and published literature on protocol deviation management (50, 51). While the deviation categories (e.g., consent errors, eligibility violations) are well-established in regulatory guidance as major deviations requiring IRB action, specific numerical thresholds (e.g., ≥3 consent errors, >10% accuracy drop) represent operationalized recommendations balancing sensitivity to systematic problems with practical IRB implementation. The AI-specific triggers reflect emerging best practices from the SPIRIT-AI extension (13) and algorithmic bias literature (35–37). Institutions should adapt these thresholds based on local regulatory requirements, trial risk profiles, and IRB capacity.

This structured mechanism enables the IRB to respond to violations in a predictable, transparent, and principled manner, fulfilling its obligations of non-harm and impartiality and reducing variability in how similar deviations are treated across protocols. In AI-enabled pediatric trials, where algorithm updates, data drift, or unanticipated performance issues can introduce new or rediscovered risks, deviation-triggered review also functions as an early-warning system that surfaces emerging safety, fairness, or consent problems before they become entrenched, helping to prevent the normalization of systemic biases affecting vulnerable children.

### Component 3: Re-consent and communication procedures

When significant errors are found in informed consent documentation or when protocol changes materially affect risks or procedures, the research content must be re-explained to affected participants or their guardians, and informed consent must be re-obtained where appropriate (56, 57). Treating informed consent as an ongoing process, rather than a one-off event, is especially important in AI-driven paediatric trials, where evolving technologies and risk profiles require structured re-consent and clear communication to maintain ethical legitimacy.

AI-specific re-consent triggers should be clearly defined to ensure continued ethical participation and regulatory compliance throughout the trial. Re-consent is required in cases of material algorithmic changes, such as when AI models are updated, retrained, or replaced in ways that meaningfully alter risk profiles, decision-making processes, or data use practices; in such instances, affected participants must be re-consented using updated IRB-approved language (56, 57). Re-consent should also be initiated upon detection of algorithmic bias. If interim monitoring identifies inequitable performance across demographic groups, participants within affected subgroups must be informed of the findings and provided the opportunity to withdraw or continue participation with full knowledge of the identified bias (56, 57). Finally, re-consent is necessary when there is an expansion of data use beyond the scope of the original consent. This includes proposals for secondary analyses, retraining of models using trial data, or data sharing arrangements not previously disclosed, all of which require prospective re-consent to uphold transparency and participant autonomy **(**56, 57).

### Component 4: continuous ethics and protocol training

The proactive model embeds continuous, scenario-based training for principal investigators and research staff into the interim review process. Following the discovery of deviations in the Taiwanese trial, the IRB mandated Good Clinical Practice (GCP)-focused retraining on eligibility assessment, documentation standards, and valid informed consent, emphasizing the practical application of ethical requirements in day-to-day workflow. Recognizing that many protocol deviations in AI-enabled trials arise from human and organizational factors rather than technical failures alone, this component institutionalizes ongoing training to build a culture of ethical awareness and procedural competence (58, 59).

AI-specific training modules should comprehensively address several core competencies. First, they should include instruction on algorithmic bias recognition and mitigation, equipping investigators to identify and address potential sources of bias in data selection, model design, and performance monitoring (58, 59). Second, they should cover digital informed consent best practices, including strategies for explaining AI systems to pediatric populations and their families in age-appropriate language while ensuring meaningful comprehension (58, 59). Third, modules should guide data governance and privacy safeguards, outlining procedures for secure data handling, implementation of access controls, and compliance with pediatric data protection standards (58, 59). Finally, training should address AI system documentation requirements, emphasizing protocols for maintaining version-controlled records of algorithm modifications, performance metrics, and deviation reports to support transparency and accountability (58, 59).

Continuous education thereby reduces the likelihood of repeated errors and supports investigators in meeting their obligations in a rapidly changing digital environment, promoting both participant protection and fair treatment of investigators, who receive guidance rather than only sanctions.

### Component 5: transparent, auditable documentation and IRB–investigator communication

The model also requires uniform, auditable documentation and open, bidirectional communication between IRBs and investigators. This includes version-controlled consent and eligibility records, periodic checks of newly enrolled participants, and secure channels for reporting emerging risks or protocol issues.

AI-specific documentation requirements should encompass several essential elements to ensure transparency, accountability, and regulatory compliance. These include maintaining algorithm version control logs consisting of timestamped records of all AI model versions, software updates, and modifications to decision-making algorithms implemented during the trial (58, 59). Documentation should also incorporate performance monitoring dashboards that provide regular reporting of AI system accuracy, calibration, and other key performance metrics, disaggregated by demographic subgroups to facilitate ongoing fairness monitoring (58, 59). In addition, comprehensive data provenance and security audit records should be maintained, detailing data sources, access controls, encryption methods, and adherence to pediatric data protection standards (58, 59). Finally, bias assessment reports should be prepared periodically to demonstrate that the AI system sustains equitable performance across age, sex, race, and socioeconomic groups, thereby supporting ethical and scientifically robust deployment (58, 59).

In this case, the IRB instituted ongoing documentation audits for all new participants and encouraged prompt reporting and discussion of any further concerns, thereby reinforcing routine quality checks and shared problem-solving. Transparent documentation and communication give substance to procedural fairness and accountability by making oversight decisions traceable and reviewable; in AI-driven pediatric trials, where data and decision processes are often complex, robust records and open IRB-investigator dialogue help ensure that corrective actions are understood, consistently implemented across sites, and feed into institutional learning and regulatory refinement over time.

### Case as a worked example

This section uses a Taiwanese AI-assisted paediatric speech-therapy trial as a worked example to illustrate how the proposed proactive, risk-sensitive interim review model operates in practice. The case is presented solely as an illustrative demonstration of the model's application in a real institutional context, rather than as an empirical investigation or data-collection study.

Methodological clarification: The normative claims in this article are derived from the conceptual analysis of ethical principles and regulatory frameworks presented above. The Taiwanese interim review is used as an illustrative case to demonstrate the application, feasibility, and potential advantages of the proposed model in one institutional setting. It does not provide empirical validation of the model's effectiveness. Future empirical research is needed to evaluate whether proactive, risk-sensitive interim review models reduce protocol deviations, improve informed consent practices, and enhance participant protection compared with standard annual oversight. The trial recruited 100 children aged 3–7 years with dysarthria to participate in an AI-assisted mobile speech-therapy program, using defined inclusion and exclusion criteria to exclude children with neurological injury, structural oral anomalies, major developmental delay, or hearing impairment. An interim review mandated by the IRB examined informed consent forms, eligibility documentation, and recruitment procedures at each trial site to assess both AI-related and human-factor risks during the study.

With respect to the first component—scheduled, risk-based interim reviews and on-site visits—the interim review itself functioned as a structured checkpoint triggered by the AI-enhanced nature of the intervention and the vulnerability of the paediatric population. Applying the AI Risk Stratification Matrix (Table 2), this trial would be categorized as moderate-to-high risk given the young age group (3–7 years), novel AI application in paediatric speech therapy, and lack of established safety data for this specific AI tool in this population. The IRB's decision to move from paper-based document review to targeted on-site visits once concerns were identified illustrates how risk-based escalation can be integrated into planned interim oversight.

The second component, structured deviation-triggered response, was activated when the interim review uncovered substantial consent and screening deviations, including incomplete or incorrectly signed guardian consent forms, inconsistent application of eligibility criteria, and documentation gaps. These findings automatically prompted site visits, full-board discussion, and a package of corrective measures, rather than being left to informal negotiation with the investigators. According to Table 3, the consent errors (≥ 3 improperly signed forms) and eligibility violations crossed explicit thresholds requiring mandatory IRB action within 7 business days.

The third component, re-consent and communication procedures, was implemented when all participants with defective consent documentation were re-contacted for renewed explanation and properly executed forms. This process ensured that ongoing participation rested on valid, auditable consent and signalled to families that deviations were taken seriously and addressed transparently.

The fourth component, continuous ethics and protocol training, was reflected in the IRB's requirement for GCP-oriented retraining for the principal investigator and research team, focusing on eligibility assessment, documentation standards, and informed consent practices. Training was framed not only as remediation for this trial but also as part of wider institutional efforts to improve ethical competence in technology-enabled studies. Finally, the fifth component—transparent, auditable documentation and IRB–investigator communication—was strengthened through the institution of ongoing documentation audits for all newly enrolled participants and the establishment of clear channels for reporting any further protocol issues. These measures aimed to normalize routine quality checks and open dialogue, turning a single episode of non-compliance into an opportunity for longer-term improvement in oversight practice.

### Actionable recommendations

The proactive, risk-sensitive interim review model proposed in this paper translates ethical principles into concrete oversight mechanisms for AI-driven pediatric trials. This section provides actionable guidance for institutional review boards, regulatory authorities, healthcare institutions, resource-constrained settings, and future policy development. These recommendations are designed to be adapted to local legal, cultural, and organizational contexts while preserving core commitments to protection, proportionality, respect for autonomy, and procedural fairness.

### Institutional review boards

IRBs should formally incorporate risk-based interim review requirements into policies for AI-driven pediatric research. High-risk protocols, those involving adaptive algorithms, vulnerable age groups, invasive procedures, or novel AI applications without established safety data, warrant quarterly or semi-annual interim reviews rather than an annual review (Table 2).

IRBs should establish explicit triggers for structured responses using the framework in Table 3: three or more informed consent errors, any ineligible enrollment, undocumented AI algorithm changes, or recurring documentation gaps automatically initiate graduated responses. Triggered audits should follow standardized procedures: investigator notification, full-board discussion, corrective action determination (training, site visits, protocol amendments), and implementation verification.

IRBs should develop systematic re-consent procedures when interim reviews identify consent documentation errors or when protocols materially change, requiring investigators to re-contact affected participants and audit completion rates. Documentation audits should examine informed consent forms, eligibility screening consistency, AI documentation (algorithm version logs, performance monitoring reports, security certifications), and adverse event reporting, conducted remotely through secure platforms when feasible. IRBs should establish secure communication channels for investigators to report emerging issues between reviews and recruit specialized expertise in AI systems and data privacy through membership, consultants, or inter-institutional networks.

### Regulatory authorities and policymakers

Regulatory bodies should issue specific guidance establishing minimum interim review standards for AI-enabled pediatric research, specifying risk-based review frequency thresholds (Table 2), required interim report content (enrollment metrics, deviation summaries, AI performance data, adverse events), and circumstances requiring expedited review. Guidance should clarify that AI system updates, model retraining, or deployment across new populations constitute protocol modifications requiring prospective IRB approval. Regulatory authorities should establish standardized triggers mandating IRB audits for AI pediatric trials (Table 3): serious noncompliance with consent procedures, systematic eligibility violations, unanticipated adverse events attributable to AI performance, privacy breach complaints, and detection of algorithmic bias. While core protective principles should be harmonized internationally, guidance must accommodate jurisdictional differences, distinguishing between mandatory requirements and adaptable practices. Regulatory bodies should provide practical implementation toolkits (risk stratification frameworks such as Tables 2, 3, interim review checklists, model informed consent language for AI trials) and sponsor training programs (online modules, webinars, certification courses) to build IRB capacity in AI ethics and digital health governance.

### Healthcare institutions and sponsors

Institutions and sponsors should allocate resources for proactive interim oversight: additional IRB meeting time, documentation audits, specialized consultants, and secure remote review infrastructure, incorporated into trial budgets prospectively. Annual GCP refresher training should be required for investigators and coordinators involved in AI pediatric trials, with mandatory modules on AI-specific risks (algorithmic bias, data drift, transparency deficits), pediatric protections, digital consent, and fairness monitoring, framed as professional development (60, 61).

Institutions should establish learning systems capturing insights from interim reviews: anonymized case repositories, periodic ethics forums, and institutional metrics tracking deviation patterns and corrective action effectiveness. Digital consent platforms, mobile applications, and AI algorithms should undergo upfront validation for security, accessibility, age-appropriate design, performance across demographic subgroups, and human oversight mechanisms before deployment in pediatric trials.

### Resource-constrained settings

Resource-constrained or smaller IRBs may lack staff time, training capacity, and technical support to implement scheduled interim reviews, deviation-triggered audits, and enhanced training at scale. This limitation argues for proportional and phased implementation rather than abandonment: the full model can initially be applied only to clearly high-risk AI pediatric trials (Table 2, High risk category), with simplified checklists, remote reviews, and shared training resources used to reduce burden while still strengthening protection.

IRBs in resource-limited settings should prioritize 1) Collaborative networks: Forming regional or inter-institutional consortia to share AI expertise, training materials, and audit resources, 2) Remote oversight tools: Leveraging secure digital platforms for document review, performance monitoring, and investigator communication to reduce travel and meeting costs, and 3) Tiered implementation: Starting with explicit deviation triggers (Table 3) that require minimal additional infrastructure, then gradually adding risk-stratified interim reviews as capacity grows.

### Priorities for future development

Professional societies and regulatory bodies should establish international forums for IRBs to share AI oversight experiences through conferences, online case repositories, and collaborative policy development. Research is needed to validate metrics assessing interim review effectiveness: timeliness of deviation detection, corrective action completion rates, consent validity, stakeholder satisfaction, and resource utilization.

As technologies evolve, guidance must address federated learning, real-world data integration, continuously learning algorithms, and generative AI applications in pediatric research. Sustainable capacity building requires professional education through graduate curricula, continuing education certificates, and mentorship programs integrating AI ethics into bioethics and clinical research training.

## Discussion

The proactive, risk-sensitive interim review model presented in this paper offers a structured framework for enhancing ethical oversight of AI-driven pediatric trials, yet several implementation challenges and contextual considerations merit careful examination.

### Implementation challenges

Resource-constrained or smaller IRBs may lack staff time, training capacity, and technical support to implement scheduled interim reviews, deviation-triggered audits, and enhanced training at scale. This is a serious limitation, but it argues for proportional and phased implementation rather than abandonment: the full model can initially be applied only to clearly high-risk AI paediatric trials (Table 2, High risk category), with simplified checklists, remote reviews, and shared training resources used to reduce burden while still strengthening protection.

A second concern is that broader use of interim reviews and automatic deviation-triggered audits could contribute to overregulation and chill valuable innovation if investigators and sponsors experience the model as unpredictable, punitive, or bureaucratic. To address this, the model must remain explicitly risk-based and learning-oriented: intensive interim oversight should be reserved for trials with significant or uncertain risks (Table 2); triggers for audits should be transparent and tightly defined (Table 3); and retraining and feedback should be framed as support for responsible innovation rather than punishment. When implemented in this way, the model can reduce avoidable harms and errors without discouraging ethically important AI research with children.

### Transferability across jurisdictions

A third challenge concerns the transferability of the model across legal and cultural contexts. Regulatory frameworks, expectations about AI transparency, and norms around digital consent differ significantly between jurisdictions, so the Taiwanese experience cannot simply be transplanted elsewhere. The model is therefore offered at the level of ethical functions—proportional interim review, structured responses to serious deviations, renewed consent when risk or procedures change, continuous ethics training, and transparent documentation and communication—rather than as a fixed procedural template. Local IRBs and regulators can adapt its components by aligning them with domestic law, calibrating thresholds and processes to local risk tolerances and capacities, and tailoring communication and consent practices to cultural norms, while preserving the core commitments to protection, respect, and procedural fairness that underlie the proposal. The risk typology (Table 1), risk stratification matrix (Table 2), and deviation triggers (Table 3) are provided as adaptable frameworks that jurisdictions can modify based on local regulatory requirements and institutional capacity.

### Methodological transparency

This paper employs conceptual normative analysis to develop a principle-based framework for AI pediatric trial oversight. The Taiwanese case is presented as an illustrative worked example demonstrating the model's practical application, not as empirical data validating its effectiveness. Future research should empirically evaluate whether proactive interim review models reduce protocol deviations, improve informed consent practices, and enhance participant protection compared to standard annual oversight. Despite these challenges, the model's functional approach—defining oversight mechanisms at the level of ethical principles rather than fixed procedures—enables adaptation to diverse settings while maintaining core participant protections. Implementation should be proportional to institutional capacity, risk-sensitive in application, and learning-oriented in spirit, balancing rigorous oversight with support for responsible AI innovation in pediatric research.

## Conclusions

This paper has developed a proactive, risk-sensitive interim review model for AI-driven pediatric trials, demonstrating that structured interim oversight grounded in core ethical principles—protection, proportionality, respect for autonomy and assent, and procedural fairness—provides a foundation for enhanced governance of evolving research technologies. The five-component model—comprising scheduled risk-based interim reviews, structured deviation-triggered responses, renewed consent procedures, continuous ethics training, and transparent documentation with open IRB–investigator communication—emerged from integration of international ethical frameworks, regulatory requirements, and practical experience from a Taiwanese pediatric AI speech-therapy trial. Together, these components function as an interdependent oversight system rather than discrete safeguards. The case example demonstrated how this integrated approach enabled early detection of consent and eligibility screening deviations, prompted targeted corrective training, and institutionalized routine oversight mechanisms. In doing so, the model transformed isolated compliance failures into opportunities for organizational learning and process improvement. To address operational implementation needs identified by reviewers, we developed three complementary tools: an AI-specific risk typology (Table 1) defining operational thresholds for distinct risk categories; a risk stratification matrix (Table 2) aligning trial risk levels with corresponding interim review frequencies; and explicit deviation triggers (Table 3) specifying mandatory IRB responses when predefined thresholds are crossed. Collectively, these tools translate proportional oversight from an ethical aspiration into a practical, consistently applicable review process. As artificial intelligence becomes increasingly integrated into pediatric clinical trials, IRBs and regulatory authorities face escalating demands for oversight mechanisms adequate to address both technological risks and persistent human-factor vulnerabilities in research practice. The functional, principle-based approach underlying the proposed model—defining oversight at the level of ethical commitments rather than fixed procedures—positions it as adaptable across diverse regulatory and institutional contexts, from resource-rich academic medical centers to resource-constrained community research settings. Implementation of proactive interim review models will require sustained commitment from institutional leaders, regulatory bodies, and research sponsors. Success depends on adequate resource allocation, cultivated expertise in AI ethics and digital health governance, and institutional cultures that frame oversight as collaborative problem-solving in service of both participant protection and responsible innovation. The model's effectiveness ultimately will be demonstrated through empirical evaluation of whether enhanced interim oversight reduces protocol deviations, strengthens informed consent practices, and builds investigator confidence in navigating AI-enabled pediatric research while maintaining the highest standards of participant protection.

## Data Availability

The original contributions presented in the study are included in the article/Supplementary Material, further inquiries can be directed to the corresponding author.
